# Celastrol induces apoptosis of human osteosarcoma cells via the mitochondrial apoptotic pathway

**DOI:** 10.3892/or.2015.4124

**Published:** 2015-07-13

**Authors:** XIAOLONG YU, XIN ZHOU, CHANGLIN FU, QIANG WANG, TAO NIE, FAN ZOU, RUNSHENG GUO, HUCHENG LIU, BIN ZHANG, MIN DAI

**Affiliations:** Department of Orthopedics, The First Affiliated Hospital of Nanchang University, Nanchang, Jiangxi 330006, P.R. China

**Keywords:** celastrol, human osteosarcoma, anticancer activity, apoptosis, mitochondrial pathway

## Abstract

Celastrol is an active compound extracted from the root bark of *Triptergium wilfordii* Hook F., also known as 'Thunder of God Vine'. It is a well-known Chinese medicinal herb that was found to inhibit tumor cell growth and promote apoptosis in several tumor cell lines. However, research into its effects on osteosarcoma cell apoptosis is still extremely limited. The present study was undertaken to determine the effect of celastrol on viability and apoptosis of osteosarcoma cells and furthermore, to illuminate the molecular mechanism of celastrol-induced osteosarcoma cell apoptosis. The 3-(4,5-dimethylthiazol-2-yl)-2,5-diphenyltetrazolium bromide (MTT) colorimetric assay was used to evaluate the viability of the cells following treatment with celastrol. The effect of celastrol on the apoptotic rate of the cells was evaluated by flow cytometry using Annexin V-PE/7-AAD staining assay. Fluorescence microscopy was used to detect the morphological changes in the human osteosarcoma U-2OS cell lines. The expression of Bcl-2 family proteins, caspase-3, caspase-8, caspase-9, cytochrome c and PARP was measured by western blotting. We found that celastrol significantly inhibited the growth of osteosarcoma cells in a dose-dependent manner, particularly U-2OS cells. Furthermore, we observed that celastrol upregulated the expression of the pro-apoptotic proteins Bax and cytochrome c and altered the ratio of Bax/Bcl-2, and triggered the mitochondrial apoptotic pathway, resulting in caspase-3 and -9 activation and PARP cleavage. To conclude, the results indicate that celastrol inhibits the proliferation of human osteosarcoma cancer cells by inducing apoptosis via the mitochondrial-dependent pathway.

## Introduction

Osteosarcoma is the most common primary malignant tumor of the bone predominantly occurring in childhood and adolescence. Among individuals younger than 20 years of age, the osteosarcoma incidence rate is 8.7 per million and the risk is higher in males (1). It usually occurs in a growing long bone such as the humerus, femur or tibia. The current treatment for osteosarcoma is neoadjuvant chemotherapy, surgical resection and chemotherapy again following surgery (2). Despite the advances in treatment options, recurrence and chemoresistance have been the main challenges confronting physicians (3–5). In patients with localized osteosarcoma, the 5-year survival rates are ~65–75% (6,7). However, the prognosis for patients with recurrence and metastases is quite poor, with 5-year survival rates ranging from 15 to 30% (8). In addition, most neoadjuvant chemotherapy drugs carry the risk of uncertain effectiveness and severe side effects, and multidrug resistant cases are common, particularly with cisplatin and doxorubicin (9). Over the past 35 years, there has been no significant improvement in chemotherapy for osteosarcoma (10). Therefore, a better understanding of the molecular mechanisms involved in osteosarcoma progression should help to explore novel therapeutic targets or develop new modalities of osteosarcoma therapy. Recently, research into osteosarcoma treatment has been focused on novel target therapies including induction of apoptosis and reduction of cell growth in osteogenic sarcoma (11–13).

Traditional Chinese medicine is an important type of complementary and alternative medicine, because it has a standardized system of diagnostics and therapies, and is practised worldwide (14). Some plant extracts used in complementary medicine, exert potent anticancer activity with low toxicity. Celastrol was found to inhibit growth and accelerate apoptosis in many human cancer cell lines such as hepatoma, breast, myeloma, pancreas and gastric cancer cell lines (15–19).

Apoptosis is a strictly controlled mechanism of cell suicide that is triggered by certain internal or external signals. It results in cell rounding and shrinkage, chromatin condensation, DNA fragmentation and shedding of smaller fragments from cells. In the intrinsic pathway, mitochondria play a central role in the occurrence of apoptosis induced by many chemotherapeutic agents (20–22). Mitochondrial membrane permeabilization, along with the collapse of electrochemical gradient across the mitochondrial membrane leads to the release of catabolic hydrolases and activators of some enzymes from the mitochondria, resulting in cell apoptosis (23,24). Bcl-2 family proteins serve as crucial regulators of this pathway through their influence on mitochondrial outer membrane permeabilization (MOMP) following homo- or hetero-association (25,26). Among Bcl-2 family proteins, pro-apoptotic proteins such as Bax, Bad and Bid, increase MOMP during apoptosis and release apoptogenic proteins into the cytosol, such as cytochrome *c*, which can bind to Apaf-1 and further activate caspase-9. Furthermore, activated caspase-9 activates downstream caspase-3 and/or -7, which in turn results in the cleavage or degradation of several key cellular substrates, including PARP, thus leading to apoptosis (27–30).

However, anti-apoptotic proteins such as Bcl-2 and Bcl-XL, can bind to activated Bax to decrease membrane permeability (31). The regulation of activated anti- and pro-apoptotic Bcl-2 family members is essential for determining the fate of cells, and disturbance of the normal apoptotic program due to alteration of the ratio by aberrant expression of these proteins may cause various apoptosis-related diseases (32,33). In addition, Bcl-2 and Bcl-XL overexpression, widely observed in various types of cancers, inhibits apoptosis and confers resistance to anticancer drugs (34,35). Therefore, induction of apoptosis through the mitochondrial-dependent pathway has been one of the targets of anticancer chemotherapy.

## Materials and methods

### Materials and reagents

Dulbecco's modified Eagle's medium (DMEM), fetal bovine serum (FBS), phosphate-buffered saline (PBS) and dimethyl sulphoxide (DMSO) were provided by Transgen (Beijing, China). A Hoechst 33258 staining kit was provided by Keygen Biotech (Nanjing, China). MTT [3-(4,5-dimethylthiazol-2-yl)-2,5-diphenyltetrazolium bromide] was obtained from Solarbio (Beijing, China). Antibodies against Bcl-2, Bax, caspase-3, caspase-8, caspase-9 and β-actin were purchased from Abcam (Cambridge, UK), and antibodies against PARP and cytochrome *c* were purchased from Cell Signaling Technology (Beverly, MA, USA). Horseradish peroxidase (HRP)-conjugated secondary antibodies were purchased from Cell Signaling Technology and Transgen. An Annexin V-PE/7-AAD apoptosis detection kit was provided by Becton-Dickinson (San Jose, CA, USA). Celastrol was obtained from Nanjing Zelang Medical Technology Co., ltd. (Nanjing, China). Stock solutions of celastrol were prepared by dissolving the celastrol powder in DMSO to a concentration of 1 M, and stored at −20°C. The working concentrations of celastrol were made by diluting the stock solution with the culture medium. The final concentration of DMSO in the medium was <0.5%.

### Cell culture

Human osteosarcoma cell lines, MG-63 (wild-type), U-2OS (wild-type) and HOS (wild-type), were obtained from the American Type Culture Collection (ATCC; Manassas, VA, USA). Cells were cultured in DMEM supplemented with 10% (v/v) FBS, 100 U/ml penicillin and 100 *µ*g/ml streptomycin. They were all placed in a humidified atmosphere containing 5% CO_2_ at 37°C. The cells used were subjected to <20 cell passages and were in the logarithmic growth phase.

### Cell viability by MTT assay

The cells were cultured in 96-well plates at a concentration of 1×10^4^ cells/well. Cell viability was determined using an MTT colorimetric assay. The cells were treated with celastrol at various final concentrations (0.5, 1, 2, 4 and 6 *µ*M, respectively), for 24, 36 and 48 h, and the control cells were treated with 0.5% DMSO. After the indicated cultivation time, 50 *µ*l of MTT (5 mg/ml in PBS) was added and the plates were incubated at 37°C for an additional 4 h. Finally, the formazan precipitate was dissolved in 100 *µ*l DMSO and the cells were shaken for 10 min. Absorbance was measured at 490 nm using a Universal microplate reader (EL800; BioTek Instruments Inc.). ELISA reader (BioTek, Model EXL800; USA). Cell growth expressed as percent viability was calculated by comparing the absorbance of treated vs. untreated cells.

### Hoechst 33258 staining of U-2OS cells

Cells were incubated with 0, 1, 2.5 and 4 *µ*M of celastrol for 48 h, harvested, fixed with 4% paraformaldehyde for 30 min at 25°C, washed 3 times with ice-cold PBS and stained with 10 mg/l Hoechst 33258 (Sigma) for 10 min in the dark at room temperature. Finally, the stained nuclei were observed under a fluorescence microscope (Olympus, ×100) with excitation at 350 nm and emission at 460 nm.

### Analysis of cell apoptosis by Annexin V-PE/7-AAD staining assay

To assess the development of apoptosis induced by celastrol, U-2OS cells were stained with Annexin V-PE/7-AAD (BD Biosciences, San Jose, CA, USA). U-2OS cells (1×10^5^) were cultured in 12-well plates. Following overnight incubation, these cells were treated with celastrol at various concentrations for 48 h and collected by trypsinization, not containing EDTA. After being twice washed with 4°C PBS, the cell pellets were suspended again in 400 *µ*l ice-cold 1X binding buffer at a density of nearly 1×10^6^ cells/ml, and then incubated with 10 *µ*l Annexin V-PE/7-AAD for 10 min in the dark at room temperature. Samples were analyzed by a flow cytometer within 1 h of staining.

### Western blot analysis

U-2OS cells were cultured in 6-well plates at a concentration of 2×10^5^ cells/well. After treatment with celastrol at various concentrations for 48 h, the cells were collected and lysed in RIPA buffer containing a protease inhibitor cocktail (Sigma Chemical, USA). The homogenates were centrifuged at 12,000 rpm for 10 min at 4°C and the supernatant fraction was collected for immunoblotting. Furthermore, protein concentrations were calculated by a BCA assay using bovine serum albumin as the standard. The same amounts of proteins were loaded and separated by electrophoresis on 12% SDS-polyacrylamide gels under a reducing condition using 100 V for 2 h. After electrophoresis, the proteins were transferred to PVDF membranes in a Tris-glycine transfer buffer using a semi-dry blotting system, and incubated with antibodies against Bcl-2, Bax, cytochrome *c*, PARP, caspase-3, caspase-8, caspase-9 and β-actin (1:1,000) overnight at 4°C. After PVDF membranes were washed in TBST 3 times, secondary HRP-conjugated antibodies were added at 1:2,000 dilution for 1 h at room temperature and the PVDF membranes were washed again in TBST 3 times. Immunoreactive proteins were detected by enhanced chemiluminescence (ECL kit; Transgen) followed by exposure to X-ray film.

### Statistical analysis

Data were analyzed using the SPSS package for Windows (version 17.0). Quantitative data are expressed as the mean ± standard deviation (SD). Statistical analysis of the data was performed using a student's t-test and ANOVA. P-values of <0.05 were considered to be statistically significant.

## Results

### Celastrol reduces the viability of U-2OS cells

**Figure 1:**
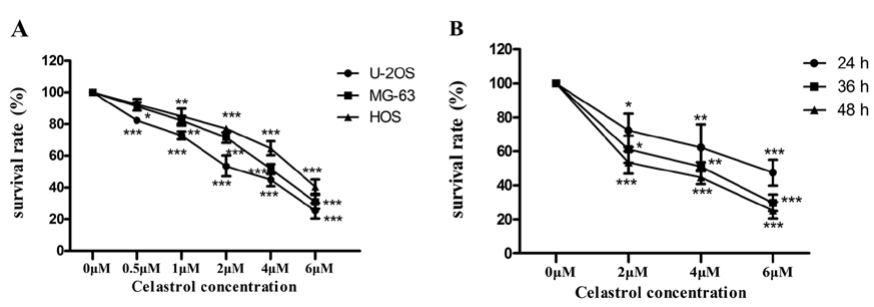
Analysis of cell viability. (A) Effect of celastrol on the viability of MG-63, U-2OS and HOS cells. Cells were treated with celastrol at different concentrations for 48 h. (B) Dose- and time-effect of celastrol on the viability of U-2OS cells. Cells were treated with celastrol at different concentrations for 24, 36 and 48 h. Cell viability was then determined and expressed as means ± SD. Significant differences from control (0 *µ*M) are indicated by ^*^p<0.05, ^**^p<0.01, ^***^p<0.001.

The effect of celastrol on the viability of osteosarcoma cell lines was determined by MTT assay, and we treated three human osteosarcoma cell lines (MG-63, U-2OS and HOS) with celastrol at different concentrations for 24, 36 and 48 h, respectively. As shown in Fig. 1A, the inhibitory effects of celastrol on the human osteosarcoma cell lines were dose-dependent, but each cell line exhibited a different sensitivity to celastrol. Obviously, the U-2OS cells were the most sensitive to celastrol. The IC_50_ value for the U-2OS cells treated with celastrol was 2.5 *µ*M at 48 h. As shown in Fig. 1B, the inhibitory effects of celastrol on the human osteosarcoma cell lines was time-dependent. Furthermore, U-2OS cells were treated with celastrol at the concentrations of 0, 1, 2.5 and 4 *µ*M for 48 h in the following assays. Our findings demonstrated that celastrol inhibited cellular proliferation in a time- and dose-dependent manner.

### Induction of morphological changes of U-2OS cells

**Figure 2:**
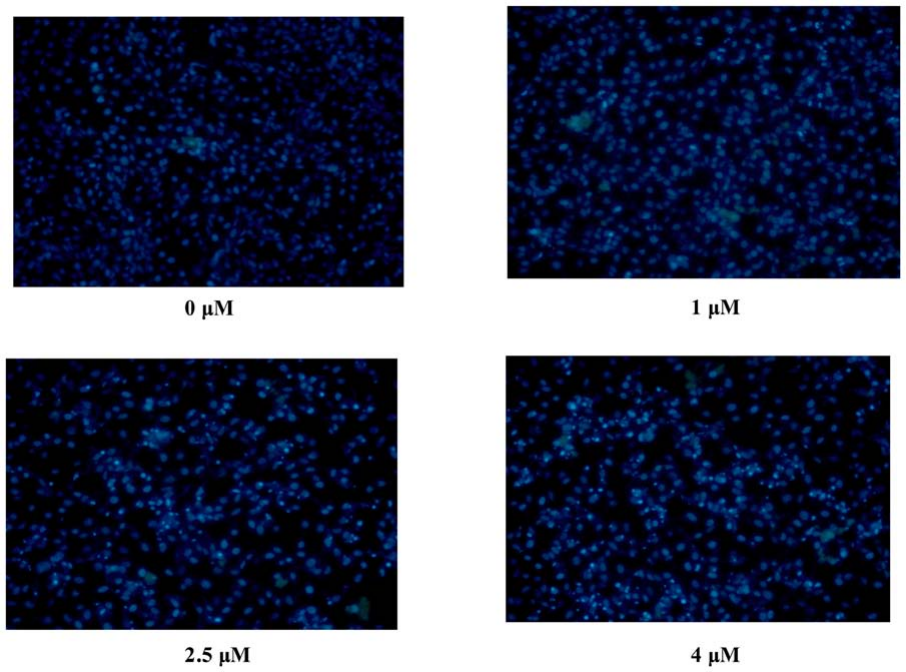
Hoechst 33258 staining of U-2OS cells. Apoptotic nuclei manifested condensed or fragmented DNA brightly stained by Hoechst 33258 (48 h) (magnification, ×100).

Untreated U-2OS cells grew well as observed by phase contrast microscopy. After 48 h of treatment, celastrol produced broken, necrotic and detached cells in a dose-dependent manner, which was consistent with the growth inhibition. Celastrol-treated U-2OS cells stained with the fluorescent DNA-binding dye Hoechst 33258 revealed condensed and fragmented nuclei, which are typical morphological features of apoptotic cells. In contrast, no morphological signs of apoptosis were observed in the untreated cells. The results indicated that cell death occurred through apoptosis (Fig. 2).

### Annexin V-PE/7-AAD staining assay

**Figure 3:**
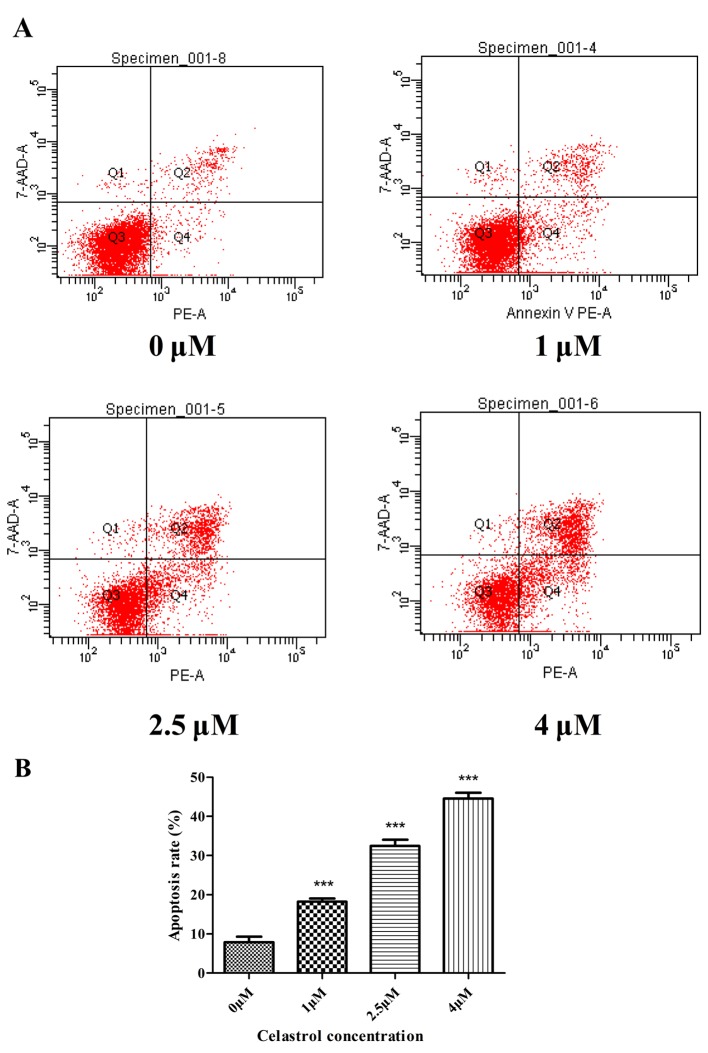
Annexin V-PE/7-AAD staining assay. (A) Representative graphs obtained by flow cytometric analysis after double-staining with Annexin V-PE/7-AAD. (B) Compared with the control cells (0 *µ*M, 7.9±1.4%), the percentage of apoptotic U-2OS cells was increased to 18.2±0.8, 32.5±1.6 and 44.6±1.4% in a dose-dependent manner after treatment for 48 h. Significant differences from control (0 *µ*M) are indicated by ^*^p<0.05, ^**^p<0.01, ^***^p<0.001.

The rate of cell apoptosis was detected by flow cytometry following double labeling with Annexin V-PE/7-AAD. Representative graphs obtained by flow cytometric analysis of the cells treated with celastrol at different concentrations for 48 h after double staining with Annexin V-PE and 7-AAD are shown in Fig. 3A. The apoptosis rate in the control cells was 7.9±1.4%. There was a dose-dependent increase in the apoptosis rate of U-2OS cells treated with celastrol. The apoptosis rates in the U-2OS cells were increased to 18.2±0.8, 32.5±1.6 and 44.6±1.4% following treatment with celastrol at 1, 2.5 and 4 *µ*M for 48 h, respectively (Fig. 3B).

### Celastrol decreases the expression of anti-apoptotic Bcl-2 and increases the expression of pro-apoptotic Bax and cytochrome c

**Figure 4:**
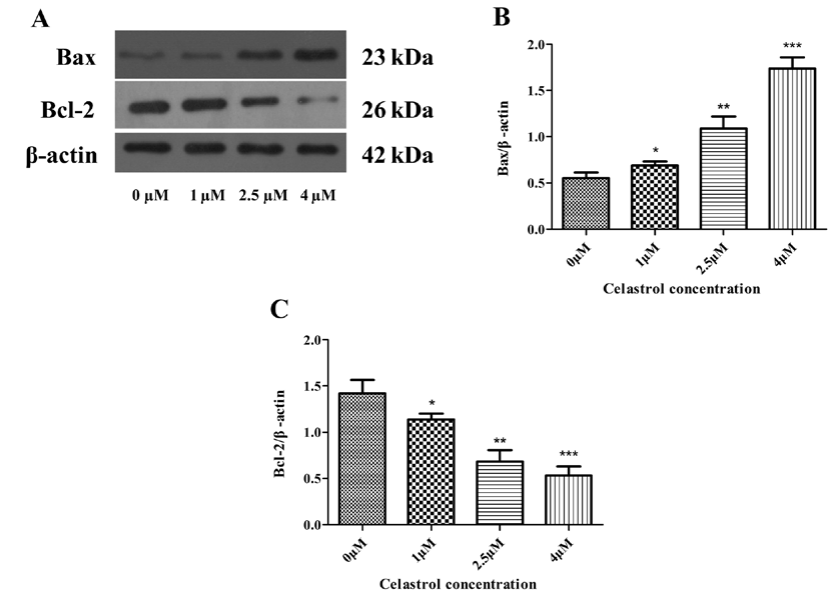
Effect of celastrol on the protein expression levels of Bcl-2 and Bax in U-2OS cells. (A) The protein expression levels of Bax and Bcl-2 were analyzed by western blot analysis. β-actin was used as the internal control for the western blot analysis. (B and C) Quantification of western blot analysis. The protein expression levels of Bax (B) and Bcl-2 (C) in celastrol-treated and control cells. The data shown are the averages ± SD (error bars), ^*^p<0.05, ^**^p<0.01, ^***^p<0.001, significant vs. control cells.

**Figure 5:**
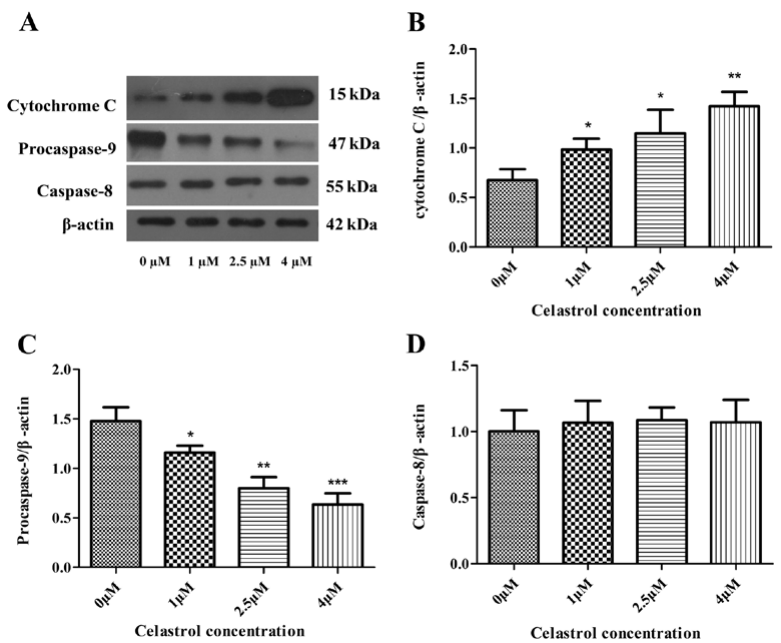
Effect of celastrol on the protein expression levels of cytochrome *c*, procaspase-9 and caspase-8 in U-2OS cells, analyzed by western blot analysis. (A) The expression levels of cytochrome *c* were significantly upregulated, while the levels of procaspase-9 were downregulated, but a change in caspase-8 expression levels was not observed. β-actin was used as the internal control for the western blot analysis. (B–D) Quantification of western blot analysis. The protein expression levels of cytochrome *c* (B), procaspase-9 (C) and caspase-8 (D) in celastrol-treated and control cells. The data shown are the averages ± SD (error bars), and significant differences from control cells are indicated by ^*^p<0.05, ^**^p<0.01, ^***^p<0.001.

To determine the molecular mechanism by which celastrol induces the apoptosis of U-2OS cells, the protein expression levels of Bcl-2 family proteins, including anti-apoptotic members such as Bcl-2, and pro-apoptotic members such as Bax and cytochrome *c*, were assessed by performing western blot analysis. The results of the western blot analysis revealed that celastrol treatment caused a profoundly marked increase in Bax proteins and the release of cytochrome *c*, and a decrease in Bcl-2 protein, when compared to these levels in the control (Figs. 4A–C and 5A and B). This demonstrates that celastrol activates the mitochondrial apoptotic pathway in U-2OS cells via regulating the expression of the Bcl-2 family proteins.

### Effects of celastrol on the expression levels of caspases

**Figure 6:**
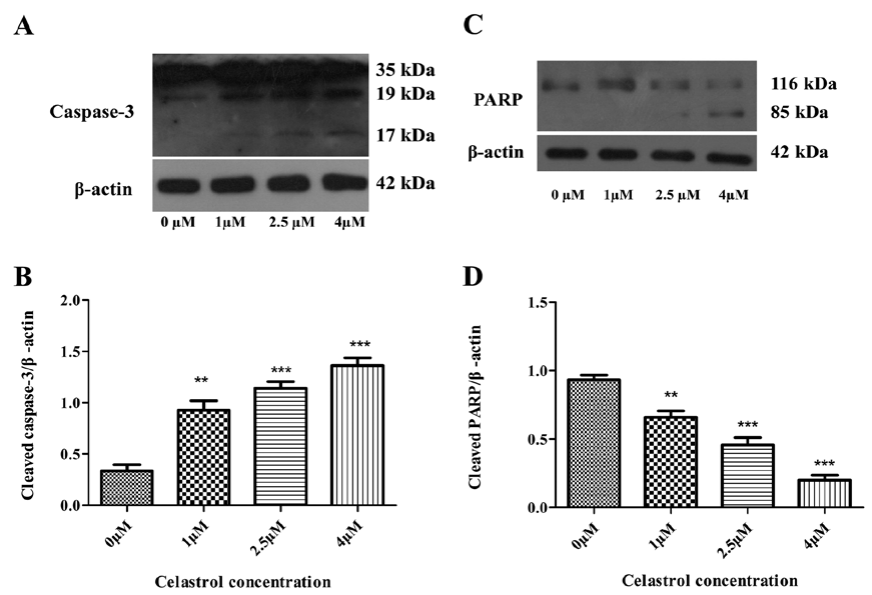
Effect of celastrol on the protein expression levels of caspase-3 and PARP in U-2OS cells. (A and C) Western blot analysis was performed and β-actin was used as the internal control. Cleavage of caspase-3 and PARP was observed. (B and D) Quantification of western blot analysis. The protein expression levels of cleaved caspase-3 (B) and cleaved PARP (D) in celastrol-treated and control cells. The data shown are the averages ± SD (error bars), ^**^p<0.01, ^***^p<0.001, significant vs. control cells.

The caspase cascade reaction is one of the most important events in the process of apoptosis through the mitochondrial pathway. Therefore, the protein expression levels of caspase-3, -8 and procaspase-9 were assessed by performing western blot analysis (Figs. 5A and 6A). Caspase-3 cleavage was observed (Fig. 6A and B), and expression levels of procaspase-9 were downregulated, both in a concentration-dependent manner as the concentration of celastrol increased (Fig. 5A and C). However, expression levels of caspase-8 were not changed in the cells treated with celastrol (Fig. 5A and D). Cleavage of PARP, a key cellular substrate, was observed (Fig. 6C and D). The results indicated that the apoptosis induced by celastrol involved the caspase cascade and was triggered through the mitochondrial pathway.

## Discussion

Apoptosis, a program of cell suicide, is an innate cellular response to eliminate abnormal or redundant cells in mammals and hence is considered an important mechanism in the action of many anticancer drugs (22). There is accumulating evidence that a wide variety of herbal medicines and compounds extracted from natural products with antitumor effects can trigger apoptosis in various tumor cells (20–22). Previous studies have demonstrated that celastrol, a triterpene extracted from the root bark of *Triptergium wilfordii* Hook F., also known as 'Thunder of God Vine, can inhibit tumor promotion (15–19). In the present study, we determined the anticancer effect and associated mechanisms of celastrol on human osteosarcoma cells lines *in vitro*. MTT results revealed that celastrol effectively suppressed the proliferation of three human osteosarcoma cell lines (MG-63, U-2OS and HOS) in a dose- and time-dependent manner. FACS analysis showed that celastrol effectively induced apoptosis in the osteosarcoma cells. Thus, we next investigated the apoptotic mechanism of celastrol on osteosarcoma cells.

Apoptosis is triggered by two different signals: the mitochondrial pathway and the cell death receptor pathway, regulated via caspase-9 and -8, respectively (36). Accumulated evidence has shown that caspases play critical roles in the apoptotic cascade. In the mitochondrial pathway (the intrinsic pathway), downstream of caspase activation is regulated by members of the Bcl-2 family. Apoptosis-associated MOMP is known to require pro-apoptotic Bax-like proteins, in the regulation of pore formation in mitochondria. Anti-apoptotic Bcl-2-like proteins in mitochondrial morphogenesis are functionally distinct from their role in apoptosis. Therefore, the ratio of Bax to Bcl-2 is vital for determining the release of many apoptogenic proteins from the mitochondrial intermembrane space, such as cytochrome *c* which can further activate caspase-9. Activated caspase-9 then activates downstream caspase-3, which causes the cleavage or degradation of various key cellular substrates, including PARP, thus resulting in apoptosis (27–30,37–39). The cell death receptor pathway (the extrinsic pathway) activates the death receptor on the cell surface (Fas/FasL) and then promotes caspase-8 activation (40–42). With this in mind, to demonstrate which signaling pathway is involved in apoptosis by celastrol, expression of Bcl-2 family proteins, caspase-3, -8 and -9 and PARP were assessed in U-2OS cells. The present data showed that celastrol-induced apoptosis was accompanied by alteration of the Bax/Bcl-2 ratio and activation of caspase-3 and -9, but not of caspase-8. Furthermore, cleavage of PARP was also observed. These findings indicated that celastrol-induced apoptosis in U-2OS cells was triggered by an intrinsic pathway.

In conclusion, we demonstrated that celastrol dose-dependently upregulates Bax expression and downregulates Bcl-2 expression in U-2OS cells. This results in the release of cytochrome *c* into the cytosol, which further activates caspase-9. Furthermore, activated caspase-9 activates downstream caspase-3 which in turn, results in the cleavage or degradation of several key cellular substrates, including PARP, and leads to the subsequent apoptosis. These results indicated that celastrol could be a potential novel therapeutic agent for the treatment of osteosarcoma. Further studies are required in order to ascertain whether celastrol can synergize with other chemotherapy drugs. In addition, studies on the *in vivo* effect of celastrol on U-2OS xenograft tumors in nude mice are in progress.
